# High Fidelity Tape Transfer Printing Based On Chemically Induced Adhesive Strength Modulation

**DOI:** 10.1038/srep16133

**Published:** 2015-11-10

**Authors:** Kyoseung Sim, Song Chen, Yuhang Li, Mejdi Kammoun, Yun Peng, Minwei Xu, Yang Gao, Jizhou Song, Yingchun Zhang, Haleh Ardebili, Cunjiang Yu

**Affiliations:** 1 Materials Science and Engineering Program, University of Houston, Houston, TX, 77204 USA; 2 Department of Mechanical Engineering, University of Houston, Houston, TX, 77204 USA; 3 Institute of Solid Mechanics, Beihang University, Beijing, 100191, China; 4 Department of Biomedical Engineering, University of Houston, Houston, TX, 77204 USA; 5 MOE Key Laboratory for Nonequilibrium Synthesis and Modulation of Condensed Matter, School of Science, Xi’an Jiaotong University, Xi’an, Shaanxi, 710049, China; 6 Department of Engineering Mechanics and Soft Matter Research Center, Zhejiang University, Hangzhou, Zhejiang, 310027, China; 7 Department of Electrical and Computer Engineering, University of Houston, Houston, TX, 77204 USA

## Abstract

Transfer printing, a two-step process (i.e. picking up and printing) for heterogeneous integration, has been widely exploited for the fabrication of functional electronics system. To ensure a reliable process, strong adhesion for picking up and weak or no adhesion for printing are required. However, it is challenging to meet the requirements of switchable stamp adhesion. Here we introduce a simple, high fidelity process, namely tape transfer printing(TTP), enabled by chemically induced dramatic modulation in tape adhesive strength. We describe the working mechanism of the adhesion modulation that governs this process and demonstrate the method by high fidelity tape transfer printing several types of materials and devices, including Si pellets arrays, photodetector arrays, and electromyography (EMG) sensors, from their preparation substrates to various alien substrates. High fidelity tape transfer printing of components onto curvilinear surfaces is also illustrated.

There are increasing interest and need for large scale integration of discrete multi-scale materials and components for the development of functional electronic systems. For example, high performance flexible and stretchable electronics, which have broad range of applications from rollable solar panels^1^,^2^, to portable electronics and to health monitoring patches^3^, require heterogeneous integration of multi scale thin high performance materials, electronics and components onto target soft alien substrates^4^,^5^,^6^,^7^,^8^,^9^. While traditional pick and place technology is not able to accomplish, transfer printing, a widely adopted approach to fabricate these high performance flexible and stretchable electronics, has proven to be a successful technology capable of heterogeneously integrating various materials, including inorganic single-crystalline dots, wires, ribbons and membranes, from a donor to a receiver substrate, such as plastic, rubber, etc^6^,^10^,^11^,^12^,^13^,^14^,^15^. During transfer printing, devices, thin ribbons, or films (referred to as ‘ink’) are first picked up from a donor substrate and then printed onto a receiving substrate, typically by using a poly(dimethylsiloxane) (PDMS) rubber stamp.

The criteria of successful pick-up and printing lie in the comparison between the adhesive strength of the stamp/ink and that of the ink/substrate^5^,^16^. If the adhesive strength between the stamp/ink is larger (smaller) than that of ink/substrate, the ink can be picked up (printed). To ensure successful and reliable picking up (printing), high (low) adhesive strength between stamp/ink is favorable. The ability to modulate the adhesive strength between the ink and stamp is of critical importance for reliable transfer printing operation. When the peeling speed of the PDMS stamp is changed, the adhesive strength alters accordingly, mainly due to PDMS’s viscoelasticity^5^,^16^. High-fidelity transfer printing could be achieved at the conditions of 1) very high adhesive strength of the PDMS stamp at high peeling speed for picking-up and 2) very low adhesive strength of the stamp at low peeling speed for printing^17^. Although the level of adhesive strength tuning can be achieved by changing the peeling speed, it requires certain instruments to accomplish accurately. In addition, the adhesive strength at an extremely low peeling speed is still relatively high^18^, which is undesirable for high fidelity printing. Some other species of materials, including thermal releasable tapes and water soluble tapes, have also been exploited for transfer printing^19^,^20^,^21^,^22^,^23^,^24^, but they typically leave certain residuals. A handful of efforts have been reported to obtain tuning through the optimization of stamp design to improve the transfer printing process^8^,^17^,^25^,^26^,^27^,^28^. However, these efforts involve costly and sophisticated multiple steps of microfabrication to obtain micro structure on the stamp.

This paper explores a simple, low-cost, high fidelity transfer printing approach based on dramatic adhesive strength modulation of commercial available adhesive tape based stamps. The adhesive strength modulation is achieved by simply introducing certain chemicals. We describe the working mechanism of the adhesion modulation, and demonstrate the application of such method by high fidelity transfer printing several different types of inks including silicon (Si) pellets, Si photodetector arrays, and serpentine shaped skin mountable EMG sensors. Furthermore, high fidelity transfer printing of inks onto curvilinear surfaces is also illustrated, where bare success can be achieved through conventional bulk PDMS stamps.

## Results and Discussions

**Figure 1:**
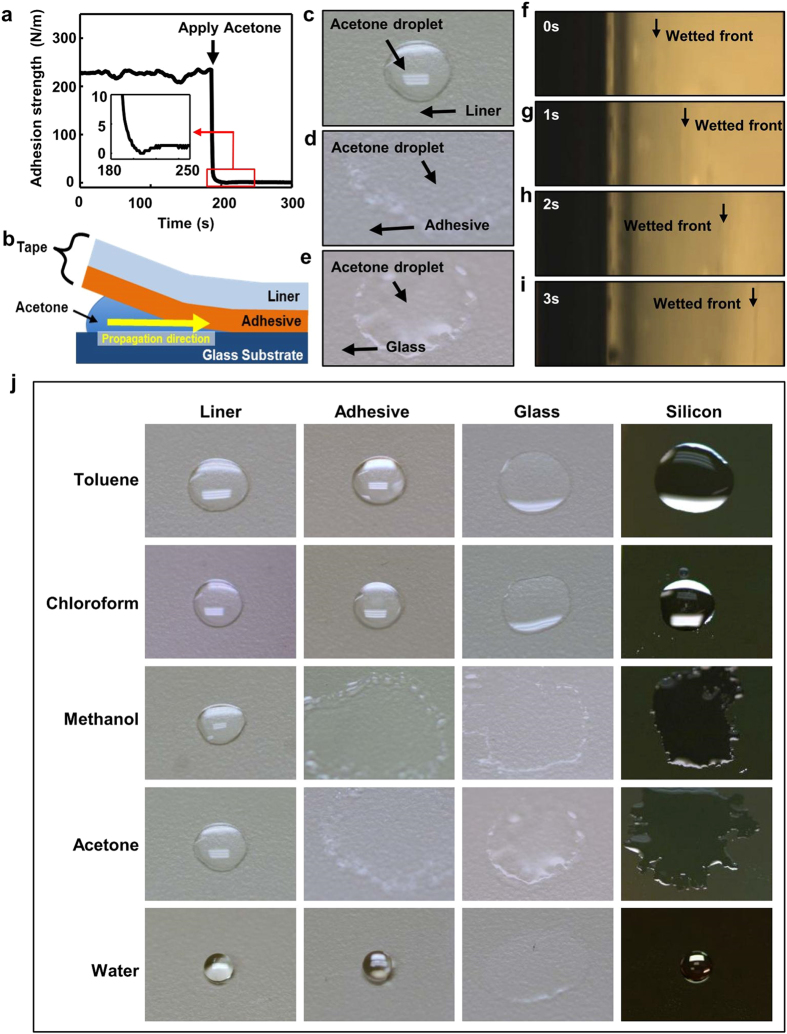
Working mechanism chemical induced tape adhesive strength modulation. (**a**) Plot of measured adhesive strength of the 3M 3850 tape before and after introducing acetone under 180^o^ peeling test. (**b**) Schematic illustration of acetone capillary propagation at the tape/glass interface. (**c**–**e**) Optical images of acetone wettability on tape liner, adhesive and glass, respectively. (**f**–**i**) Optical images of acetone propagation at interface between the adhesive and glass at 0s, 1s, 2s, and 3s, respectively. (**j**) Optical image Illustration of wettability of multiple solvents on the 3M 3850 tape liner, adhesive, glass and Si wafer. Strong wettability of methanol and acetone on adhesive, glass and Si explains the ability of chemical induced adhesive strength modulation.

In this study, we have developed a method to modulate the adhesive strength of commercially available adhesive tape to pick-up and print materials and devices with high yield. Typically, the adhesive strength of 3M adhesive tapes have adhesive strength much higher than that of PDMS (Sylgard 184, ratio of monomer to crosslinker is 10:1). For example, the adhesive strength between 3M 3850/glass is about 220 N/m, which is more than 20 times higher than that of PDMS/glass^18^. High adhesive strength ensures high fidelity pick up of the inks from the donor substrate, as illustrated in the following examples. In addition, the adhesive strength of the tape decreases significantly upon the introduction of acetone, resulting in high-fidelity printing of ink onto receiving substrates. Figure 1a shows the adhesive strength of the 3M 3850 tape on glass before and after introducing acetone under 180^o^ peeling tester (ESM301, Mark-10 Corp.) at the peeling speed of 1 mm/minute. A significant drop of adhesive strength from 220 N/m to almost 0 N/m was obtained as soon as acetone is introduced. Details of the test are illustrated in Supplementary Fig. S1 online.

Whether the tape’s adhesive strength can be modulated is determined by the solvent wettability on the tape and the substrate. As shown in Fig. 1b, the tape consists two layers, i.e. the adhesive and liner. The wetting properties of acetone on the adhesive, liner, and the glass substrate are show in Fig. 1c–e. Acetone has high wettability on the adhesive and the glass, but low wettability on the liner. Once acetone is introduced, the interface between adhesive/glass (Fig. 1c) will then be easily wetted, resulting in delamination of the tape from the glass. Figure 1f–i show the automatic wetting propagation at the interface between the tape/glass over time, explaining why the adhesive strength dropped to almost 0 N/m. High wettability on both the adhesive and inks are required for dramatic interfacial adhesive strength reduction, and thus a successful transfer printing.

To further examine the working mechanism, different types of solvents including toluene, chloroform, methanol, and deionized (DI) water were studied. Figure 1j includes images of the different solvents on 3M 3850 tape liner, adhesive, glass, and silicon. Figure 1j indicates that methanol and acetone has the highest wettability (i.e. the lowest contact angle) on the adhesive, glass, and silicon. As summarized in Supplementary Table S1 online, only methanol and acetone work well for the adhesive modulation for two types of tapes (3M 3841 and 3850). For other solvents, as shown in Fig. 1j, the wettabilities on adhesive are low, and the adhesive strength at the interface was not reduced. In this study, 3M 3850 tape was primarily used as the stamp and acetone as the adhesive modulator. However, this process is applicable to other different types of tapes and multiple solvents, such as 3M 3841 and methanol.

**Figure 2:**
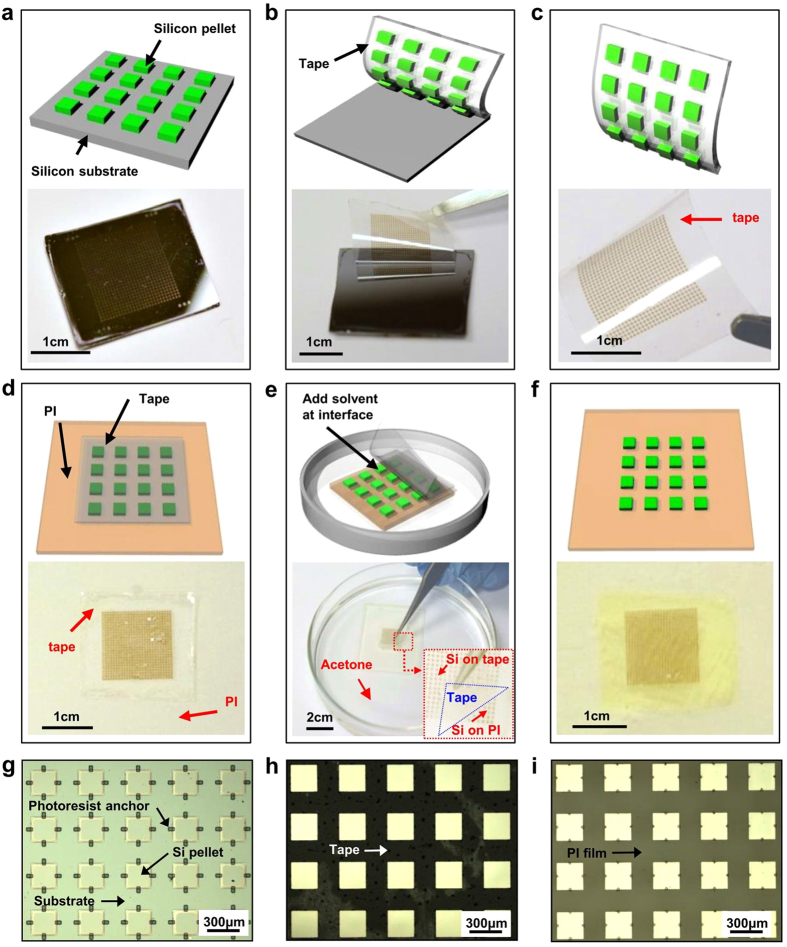
TTP operation procedure. (**a**) Fabricated Si pellets array on an SOI wafer. (**b**) Picking up Si pellet array through peeling off the tape. (**c**) The high yield picked up Si pellet array on the tape. (**d**) Laminating the tape with Si pellet array facing the PI mediate and substrate. (**e**) Introducing acetone and peel off the tape from substrate. (**f**) The high yield printed Si pellet array on the PI. (**g–i**) Optical images of Si pellet array on SOI wafer, tape and PI, respectively. No variation of spatial configuration existed during the TTP operation procedure.

To demonstrate its application for high fidelity transfer printing, ultra-thin silicon pellet arrays, as inks, were prepared and transfer printed from their mother wafer to thin polyimide (PI) substrates. Figures 2a–f show the transfer printing process, with the schematic images on the top and optical images on the bottom. Si pellet arrays were harvested from silicon-on-insulator (SOI) wafers with 1.25 μm thick top Si layer. The preparation included defining and isolating the Si pellets by standard photolithography and reactive-ion etching (RIE), followed by etching down of SiO_2_ in buffered oxide etchant (BOE 1:6), and anchoring the pellets by photoresist AZ 5214 after photolithography and patterning. The Si pellets were then fully immersed in concentrated hydrofluoric acid (HF, 49%) to remove the remaining SiO_2_, thus inducing complete undercut etching, which ensures that the Si pellets are anchored only by the photoresist on the edges. Details of the fabrication steps and transfer printing are illustrated in Supplementary Fig. S2 online. Figure 2a shows the Si pellets on an SOI wafer. A 3M 3850 tape is then brought into contact with the wafer. Due to its strong adhesive strength, the tape picks up all the Si pellets by peeling off to crack the photoresist anchors, as illustrated in Fig. 2b. Figure 2c shows that the Si pellet array on the tape was held by a tweezer. The printing of the Si pellets from the tape onto a receiving substrate of PI was carried out by first spin coating a 1-μm-thick PI (PI2545, HD Microsystems) on a temporary glass substrate; half curing at 110 °C for 20 seconds, and laminating the tape with the Si pellets facing the PI layer, as shown in Fig. 2d. Fully curing the PI to solidify ensures a strong interfacial adhesion between the Si pellets and the PI. When immersed into acetone, the tape loses adhesion and simultaneously separates from the Si pellet array and the PI substrate (Fig. 2e). Figure 2f shows 729 Si pellets (an array of 27 × 27) printed on the PI, with a yield of 100%.

The TTP approach was further examined to ensure its high fidelity. As shown in Fig. 2g, the array of Si pellet (250 μm × 250 μm) on the SOI wafer is separated by 150 μm in both x and y directions. The pellet arrays maintain the same configuration after being picked up on the tape (Fig. 2h) and printed on the PI (Fig. 2i). No residual from the tape was observed. The significant decrease of the interfacial adhesive strength renders the TTP a high yield, high fidelity, and viable technique.

**Figure 3:**
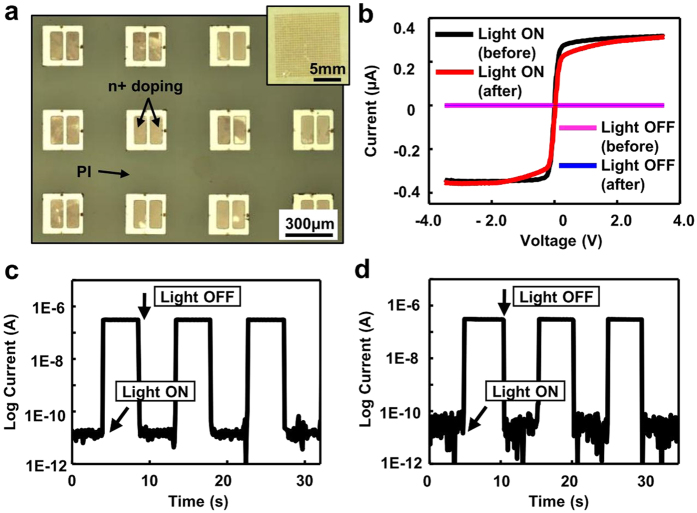
TTP of Si photodetectors. (**a**) Optical image of Si based photodetector array on PI after TTP. The inset is optical image of the entire array. (**b**) Current-voltage curves of the photodetector under illumination and dark before and after TTP. (**c,d**) Dynamic photoelectrical responses of the photodetector before and after TTP, respectively.

In order to ensure that there is no damage or disruption in the ink during such TTP process, ultra-thin (1.25 μm) Si based photodetectors, as representative devices, were prepared, transfer printed, and characterized. The major preparation process involved selective solid state diffusion doping to define the active photodetector region, device isolation, photoresist anchoring, and undercut etching. The detailed steps for the preparation of photodetector array are schematically illustrated in Supplementary Fig. S3 online. The photodetectors are configured with two back-to-back photodiodes (n-p-p-n). Figure 3a show optical images of the high yield transfer printed Si photodetector array on PI, with each device having a size of 250 μm × 250 μm. The photoelectrical response of the photodetector on the PI substrate is shown in Fig. 3b–d. Under illumination the photocurrent was 0.31 μA and the dark current was 48 pA at 3 V bias. The calculated current ratio under illumination and dark is ~6.4 × 10^3^ at 3 V bias, based on the equation *R* = *I*_*bright*_*/I*_*dark*_. The dynamic photo responses of the device before and after TTP were also characterized by measuring the current at a constant bias of 3.5 V with the light on/off in a cyclic manner. As shown in Fig. 3c–d, no significant performance difference was observed before and after transfer printing. The device on the thin tape experienced negligible strain when being picked up. Upon printing onto the new substrate, the device was left completely intact and undisturbed by the inert tape, because the adhesive strength between the tape and devices was so severely reduced upon application of the solvent.

**Figure 4:**
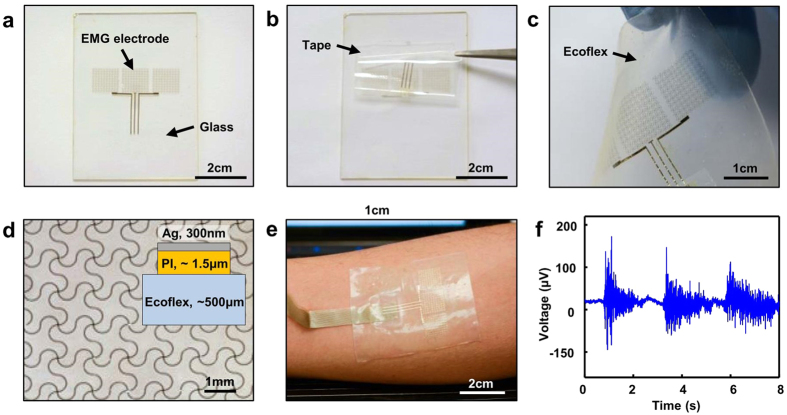
TTP of a skin mountable EMG sensor. (**a**) Optical image of a fabricated EMG sensor on glass. (**b**) Picking up the EMG sensor through the tape. (**c**) EMG sensor printed on stretchable Ecoflex substrate. (**d**) Optical image of the serpentine shaped filamentary stretchable electrode as component of the sensor. The inset is schematic cross-section of the sensor. (**e**) An EMG sensor mounted on the skin of forearm for measurement. (**f**) A representative example of recorded EMG signal during the muscle contraction and relaxation.

To further demonstrate its capability, a skin mountable, stretchable epidermal EMG sensor was fabricated through TTP. The EMG sensors employs serpentine shaped filamentary thin electrodes to enable its mechanical stretchability, which has been reported elsewhere^21^,^22^,^24^,^29^. The details of the fabrication are schematically illustrated in Supplementary Fig. S4 online. Figure 4a shows an optical image of a fabricated representative EMG sensor on a temporary glass substrate. Following similar transfer print procedure, the EMG sensor was picked up by the tape (Fig. 4b) and printed onto a soft elastomeric silicone substrate (Ecoflex, Reynolds Advanced Materials). A thin, stretchable EMG sensor is shown in Fig. 4c. The serpentine filamentary stretchable electrodes (Fig. 4d) maintained their geometrical configurations after the transfer printing. To complete the sensor, the electrodes were connected with anisotropic conductive film (ACF, Elform Heat Seal Connectors) ribbon tables, interfacing with a 136-channel Refa amplifier (Twente Medical Systems International, The Netherlands). Then the completed EMG sensor was mounted on the superficial muscles of the anterior forearm for measurement (Fig. 4e). A two-second isometric wrist flexion followed by a one-second relaxation was performed repetitively. Figure 4f is a representative demonstration of the recorded EMG signals. Muscle contractions and relaxations can be observed and differentiated through their EMG patterns.

**Figure 5:**
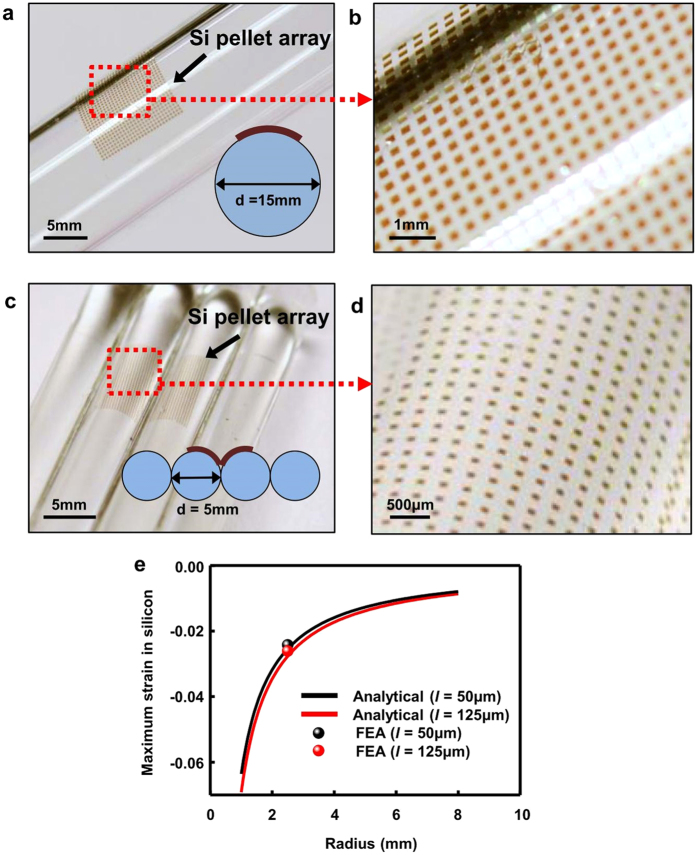
TTP on curvilinear surfaces. (**a,b**) Si pellet array on the surface of a cylindrical tube with diameter of 15 mm. (**c,d**) Si pellet array on concave surface from two adjacent cylindrical tubes with diameter of 5 mm. (**e**) Analytical and FEA results of the maximum strain in Si versus the bending radius of the tape.

Besides its ability to dramatically modulate the adhesive strength to enable high fidelity transfer printing, the thin and flexible nature of the tape relative to conventional thick PDMS stamp also offers a capability to conform to curvilinear surfaces, such as convex or concave surfaces. Conventional transfer printing typically involves using a thick PDMS stamp. Therefore significant amount of strain might be imposed onto the devices upon conforming to curved surfaces, which directly results in damage, especially in those based on brittle materials. Using TTP, devices such as those described above would be able to be printed on curvilinear surfaces without experiencing high level of strain. Fig. 5a–b show optical images Si pellet array printed onto the convex surface of a glass cylinder with thin layer of silicone coated on. Transfer printing yield of 100% was easily achieved without cracking the Si pellets. Besides on simple convex surfaces, Si arrays were also transfer printed onto concave surfaces from two adjacent convex surfaces (Fig. 5c–d).

To quantitatively understand the associated strain in the devices during TTP onto curvilinear surfaces, analytical and finite element studies were performed. A two-dimensional finite element model was established to study the deformation of the device. Plane-strain element (CPE4) in the finite element software ABAQUS was used. Supplementary Fig. S5a online shows a schematic model of a 3M 3850 tape/ink with liner (polypropylene) layer on the top, adhesive (polyurethane) layer in the middle and Si pellet on the bottom. The half-length of Si pellet is denoted by *l*. The thicknesses are *h*_*s*_ = 80μm, *h*_*a*_ = 30μm and *h*_*f*_ = 1.25μm (or 0.3μm), where the subscripts of *s*, *a,* and *f* stand for polypropylene, adhesive, and Si, respectively. The Young’s moduli and Poisson’s ratios are *E*_*s*_ = 350MPa and ν_*s*_ = 0.42 for polypropylene^30^, *E*_*a*_ = 2MPa and ν_*a*_ = 0.49 for polyurethane^31^, and *E*_*f*_ = 130GPa and ν_*f*_ = 0.3 for Si^32^. A rotation which is equal to the length of Si divided by the radius of bending curvature is applied at the two ends of the model. Supplementary Fig. S5b-c online show the strain contours of the tape and Si. The soft adhesive serves as a strain isolation layer to reduce the strain in Si pellet significantly and thus protect it from fracture. An analytical mechanics model is also established to predict the maximum strain in Si pellet to provide design guidelines for TTP. The strain in Si pellet is the summation of the bending strain and membrane strain. Because the bending rigidity of Si pellet is much smaller in comparison with the other two layers and the polyurethane is very soft, the maximum bending strain can be approximated by


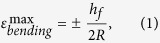


where *R* is the radius of bending curvature. The membrane strain can be obtained by modeling polypropylene as an elastic beam and polyurethane as a shear lag^33^. The maximum membrane strain is given by


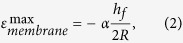


where 
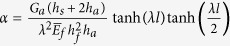
 with 
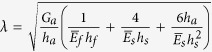
, *G* is the shear modulus and 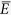 is the plane strain modulus. The maximum strain in Si pellet is then given by


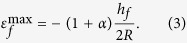


Figure 5e shows well matched results of the maximum strain in Si pellets as the function of the bending radius, from both the FEA and analytical model predictions. Through using such tape transfer, the maximum strain in the Si is far below its fracture strain (~1%).

## Conclusions

The TTP presented in this paper represents a simple, powerful, and high fidelity approach to heterogeneously integrating materials and devices onto substrates based on adhesive strength modulation of the tape stamps. The mechanism of chemical induced adhesive strength modulation suggests routes that have not been explored in conventional transfer printing and other related areas. Though only a few stamps and “poisoning” solvents have been identified, a broader range of stamp materials and modulation scenarios including chemical, physical, electrical, etc. would also be possible. The results described in this paper demonstrate the feasibility of TTP for fabricating broad range of devices. This approach can be further extended to high fidelity transfer printing of layered 2-Dimensional nanomaterials such as graphene or transition metal dichalcogenides.

## Methods

### Si pellet array fabrication

Ultra-thin Si pellet arrays were fabricated using an SOI wafer with 1.25 μm thick top layer of single crystal Si. The SOI wafers were cleaned using acetone, IPA and DI water and then baked it at 110^o^C for 2 minutes on a hotplate for dehydration. The Si squares of 250μm × 250μm were formed using photolithography and reactive ion etching (RIE). The wafer was immersed in buffer oxide etchant (BOE, 1:6) for partial undercut etching for 15 minutes. Photoresist (PR) anchor was formed by spin coating and photolithography to prevent future floating of Si during following undercut releasing. The Si pellets are then fully immersed in concentrated hydrofluoric acid (HF, 49%) for two hour to remove the remaining SiO_2_ thus inducing completely underneath etching.

### Si photodetector fabrication

Ultra-thin Si based photodetectors were fabricated using an SOI wafer with the top layer of 1.25 μm thick single crystal Si. The main fabrication steps involve selective doping to create active regions and harvesting the thin devices through sacrificial undercut etching of buried oxide. Similar approach has been reported elsewhere^34^. Specifically, 600 nm thick SiO_2_ doping mask was formed on a SOI wafer using spin on glass (700B, Filmtronics) and patterned based on standard photolithography and etching. Because the SOI wafer is slightly doped as p-type (resistivity: 11.5 Ω cm), phosphorous based spin-on-dopant (P510, Filmtronics) was used for the doping process at 950 °C to form two back to back n-p-p-n diodes. The top Si device was patterned into 250 μm × 250 μm square arrays by reactive ion etching (RIE) using sulfur hexafluoride (SF_6_) gas. The photodetector was anchored by photoresist to prevent floating away in hydrofluoric acid (HF, concentration 49%) and followed by SiO_2_ undercut etching process, similarly to the process described in the Si pellet fabrication.

### EMG sensor fabrication and transfer printing

To prepare EMG sensors, a glass slide was cleaned by acetone, IPA, and DI water and then baked it at 110 °C for 2 min on hotplate for dehydration. The PI precursor solution was then coated on the glass by spin-casting and the film was cured at 250 °C for 1 hour. 300 nm of Ag layer was deposited on the PI by e-beam evaporation. The electrodes were patterned through photolithography and wet etching. The PI, as supporting structure, was patterned by RIE under O_2_ plasma, using the metal electrodes as mask. Ag/PI structured EMG sensor was picked up from glass substrate by using the tape 3M 3850. Before transfer printing, thin SiO_2_ (50nm) layer was deposited on the tape by e-beam evaporation. A thin Ecoflex (~500 μm) was spin coated on glass substrate and cured at 90 °C for 5 minutes. Thereafter, the Ecoflex film was exposed by ultraviolet ozone (UVO) treatment to generate hydroxyl terminate group on the Ecoflex film surface. The tape was laminated onto Ecoflex substrate and heated at 70 °C for 10 min to form strong covalent bonding between the SiO_2_ on PI and Ecoflex substrate. To retrieve the tape, the sample was immersed in acetone, and then the tape delaminated immediately from substrate, with the EMG sensor remaining on the thin Ecoflex. ACF, in the form of thin ribbon cables, attached to electrode pads of the mesh at one side and to a printed circuit board (PCB) at the other, provides electrical connections to external amplifier for signal acquisition. To cure the ACF at 170 °C on a flat iron for 1 minute forms robust mechanical joints at these points of connection. The EMG sensor with ACF cable bonded was finally peeled off from the glass substrate carefully.

### TTP onto curvilinear surfaces

Curvilinear surfaces that have different radii of curvature were cleaned and coated with thin (thickness 100 μm) Ecoflex. After picking up the Si pellet array, the tape was conformably attached to the curvilinear surface and the transfer printing process was followed.

### Measurement of EMG signals

The measurements of EMG signals were conducted under the approval from the Institutional Review Board (Protocol number: 14139-01) at the University of Houston. All experiments were performed in accordance with relevant guidelines and regulations. The subject is a co-authors in the paper. Research was carried out with informed signed consents from the subject.

## Additional Information

**How to cite this article**: Sim, K. *et al.* High Fidelity Tape Transfer Printing Based On Chemically Induced Adhesive Strength Modulation. *Sci. Rep.* **5**, 16133; doi: 10.1038/srep16133 (2015).

## Supplementary Material

Supplementary Information
