# Supplementary material for: High Fidelity Tape Transfer Printing Based On Chemically Induced Adhesive Strength Modulation
Source: Sci Rep. 2015 Nov 10;5:16133. doi: 10.1038/srep16133 (PMC4639845; doi:10.1038/srep16133)
Supplement: Supplementary Information [file srep16133-s1.docx]

Supplementary Information

**High Fidelity Tape Transfer Printing Based On Chemical Induced Adhesive Strength Modulation**

Kyoseung Sim^1,†^, Song Chen^2,†^, Yuhang Li^3^, Mejdi Kammoun^2^, Yun Peng^4^, Minwei Xu^5^, Yang Gao^2^, Jizhou Song^6^, Yingchun Zhang^4^, Haleh Ardebili^2^, & Cunjiang Yu^1,2,7,*^

^1^Materials Science and Engineering Program, University of Houston, Houston, TX, 77204 USA

^2^Department of Mechanical Engineering, University of Houston, Houston, TX, 77204 USA

^3^Institute of Solid Mechanics, Beihang University, Beijing, 100191, China

^4^Department of Biomedical Engineering, University of Houston, Houston, TX, 77204 USA

^5^MOE Key Laboraory for Nonequilibrium Synthesis and Modulation of Condensed Matter, School of Science, Xi’an Jiaotong University, Xi’an, Shaanxi, 710049, P.R.China

^6^Department of Engineering Mechanics and Soft Matter Research Center, Zhejiang University, Hangzhou, Zhejiang, 310027, China

^7^Department of Electrical and Computer Engineering, University of Houston, Houston, TX, 77204 USA

^†^ These authors contributed equally to this work.

* cyu15@uh.edu

**Table 1S** Summary of different types of solvents and tapes for tape transfer printing.


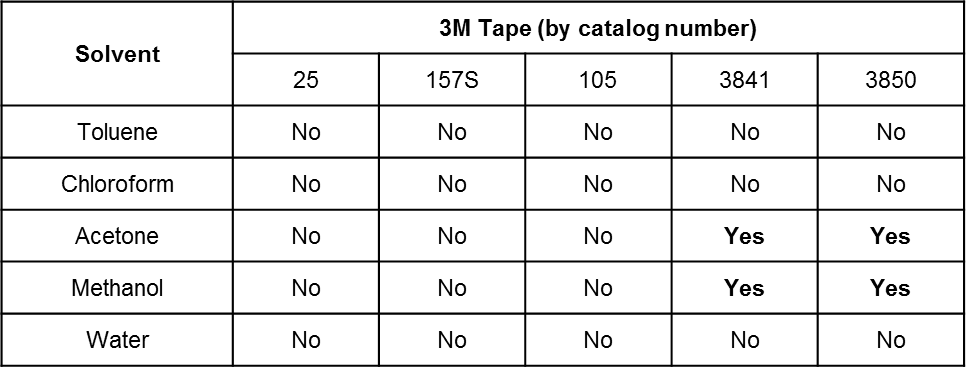


**
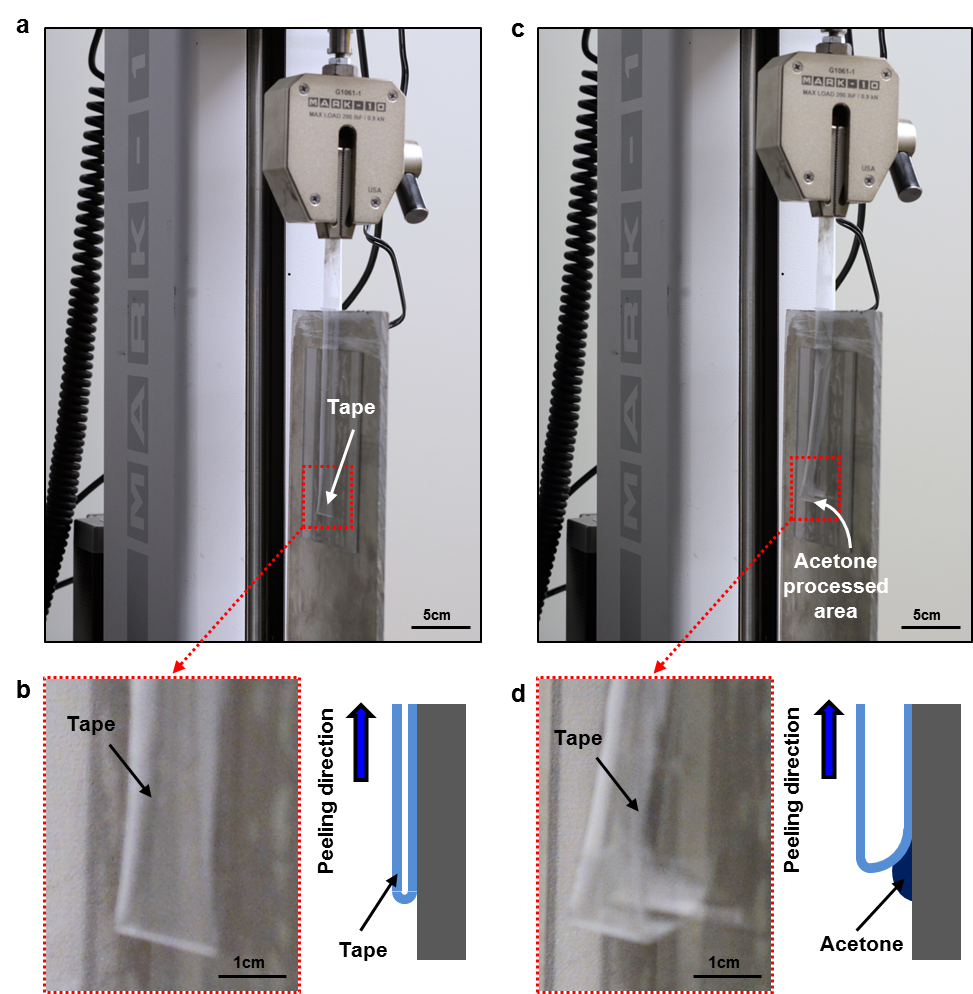
**

**Figure S1.** The illustrations of peeling test. (a), (b) before and (c), (d) after applying acetone.

**
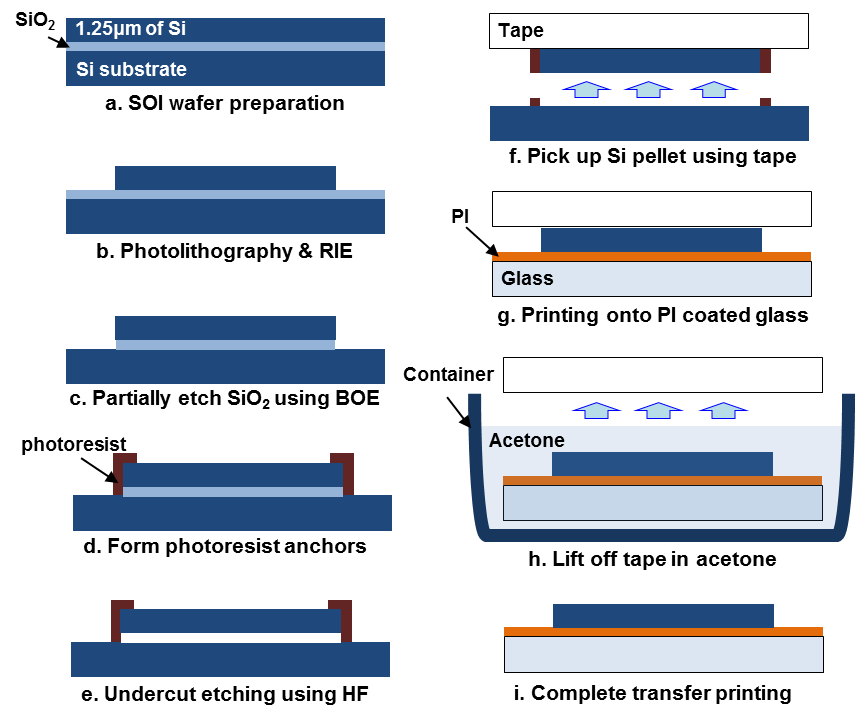
**

**Figure S2.** Schematic processes for fabricating Si pellet array and printing them onto a PI substrate using TTP.

**
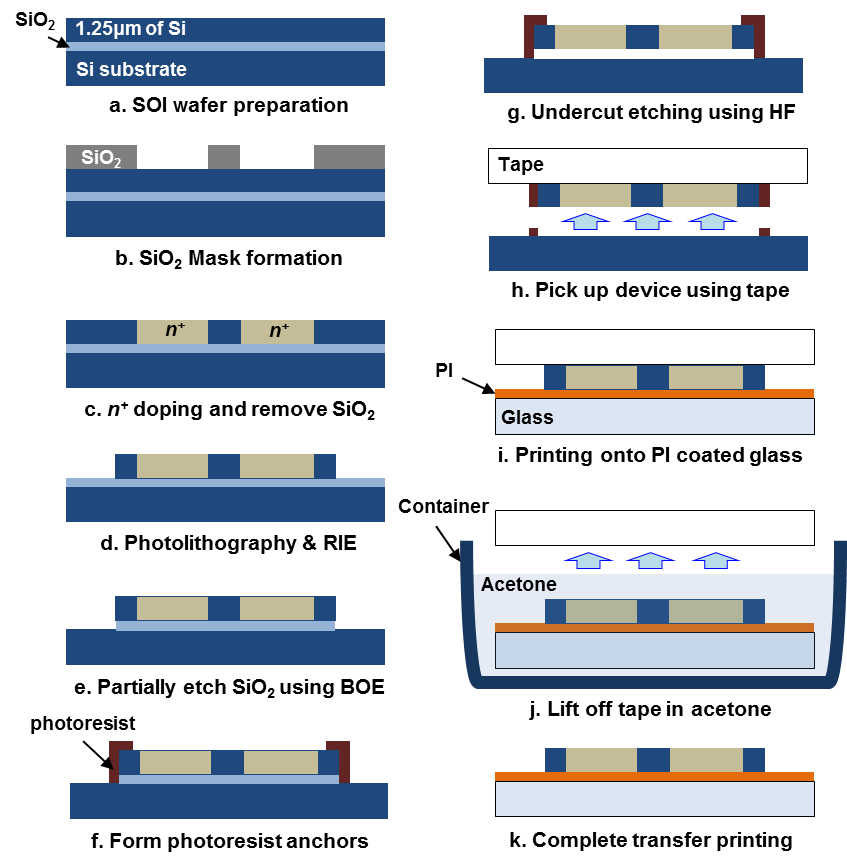
**

**Figure S3.**  Schematic processes for fabricating Si photodetector array and printing them onto a PI substrate using TTP.

**
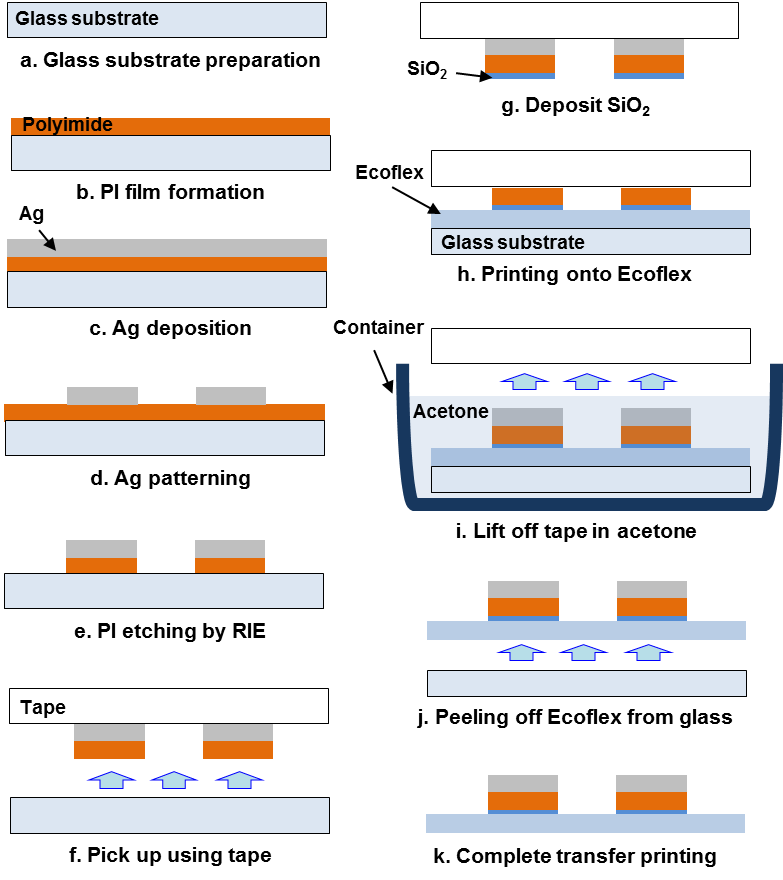
**

**Figure S4.** Schematic fabrication process for a skin mountable epidermal EMG sensor.

**
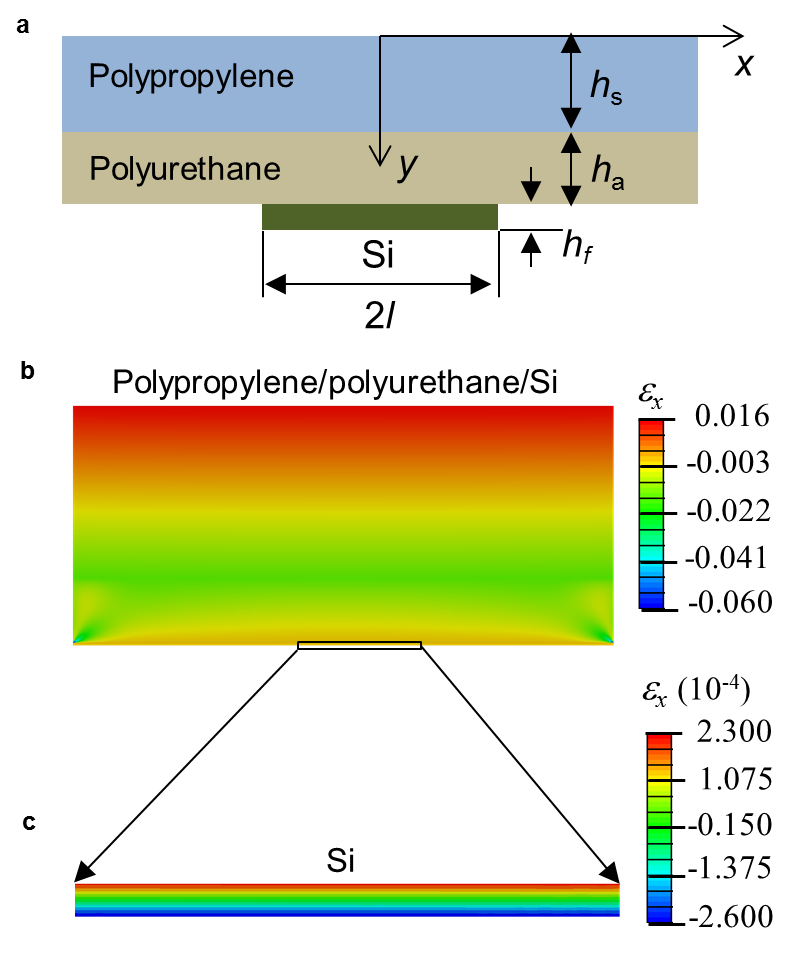
**

**Figure S5.** (a) Schematic illustration of the modeled system. (b,c) FEA strain contours of the tape and Si (*l*=125μm), respectively, while bent with a bending radius of R = 2.5mm.
